# Clonal spread of carbapenemase-producing *Enterobacteriaceae* in a region, China

**DOI:** 10.1186/s12866-022-02497-y

**Published:** 2022-03-29

**Authors:** Changfu Yin, Weiwei Yang, Yuanpeng Lv, Peng Zhao, Jiansheng Wang

**Affiliations:** 1 The Experimental Center, Clinic College of Hebei Medical University, 309 South Jianhua Street, Shijiazhuang, 050031 Hebei China; 2 Clinical Laboratory, Hebei General Hospital, 348 Hepingxi Road, Shijiazhuang, 050051 Hebei China

**Keywords:** Carbapenemase-producing, *Enterobacterales*, Patient transfer, Infection control, *Wzi*, ST11

## Abstract

**Background:**

The increasing number of carbapenemase-producing *Enterobacterales* (CPE) has become a serious problem globally. This study aimed to elucidate their geographically epidemiological characteristics.

**Methods:**

Resistance genes were identified by polymerase chain reaction (PCR) and sequencing. Bacterial genotyping was studied using multilocus sequence typing (MLST) and *wzi* typing. The transferability of carbapenemase genes was determined by a broth mating method. The relationships between the rates of antimicrobial consumption and the prevalence of CRE were performed by Pearson's or Spearman's correlation analyses.

**Results:**

A total of 930 phenotypically confirmed carbapenem-resistant *Enterobacterales* (CRE) isolates collected from 19 hospitals were genotypically characterized. *K. pneumoniae* (KP) and *E. coli* isolates were 785 (85.14%) and 96 (10.41%) among 922 CPE isolates. Two major carbapenemase genes *blaKPC-2* and *blaNDM* in CPE isolates accounted for 84.6% (*n* = 780) and 13.77% (*n* = 127). ST11 comprised 86.83% (633/729) of KPC-2 KP isolates. Different combinations of extended spectrum-β-lactamase (ESBL) genes of *blaSHV*, *blaCTX*, and *blaTEM* were found in KPC-2 producing KP isolates, and *blaCTM-M-14/15*, *blaSHV-11/12* and *blaTEM-1* were common ESBL genotypes. The *wzi* typing method could further subdivide ST11 KP group into at least five subgroups, among which *wzi*209 (69.83%, 442/633) was the most frequently isolated, followed by *wzi*141 (25.28%, 160/633). Conjugation assays showed that high conjugation rates were observed in CPE (15.24%, 32/210) for NDM plasmids, but relatively low (8.1%, 17/210) for KPC-2 plasmids. Different STs, different *wzis* and temperature could influence plasmid conjugation efficiency. No associations between the rates of antibiotics consumption and CPE prevalence were observed. The number of intra-hospital and inter-hospital transfers of CPE patients increased gradually from 18 (17.82%, 101) and 12 (11.88%, 101) in 2015 to 63 (30.73%, 205) and 51 (24.88%, 205) in 2018 (*p* = 0.016 and *p* = 0.008), respectively. Evidence-based measures could effectively reduce the prevalence of ST11-*wzi*209 clone but failed to control the dissemination of ST11-*wzi*141 KP clone.

**Conclusions:**

Clonal spread of CPE, especially KPC-2 ST11 KP was the key factor contributing to the CPE increase in the region. Continued vigilance for the importations should be maintained. Coordinated regional interventions are urgently needed to reduce CPE threat.

## Background

Owing to the consequences of unreasonably excessive and frequently unnecessary use of antibiotics in humans and animals, bacterial strains that harbored novel and transmissible antibiotic resistance genes were continually emerging [1]. Of these, carbapenemase-producing *Enterobacterales* (CPE) strains were of particular concern as they possessed an extremely high potential for transmission and had been associated with increased mortality, longer hospital stays and higher hospital costs [2]. This scenario had resulted in the introduction of tigecycline, colistin and ceftazidime-avibactam as the novel therapeutic agents for these infections. However, the extensive use of these antibiotics in clinical settings was rapidly followed by the emergence of new resistance to these drugs [3–5], leaving treatment options even fewer or non-existent. Given the paucity of novel or clinically proven effective antibiotics, infection control and prevention must be given an urgent priority.

In response, numerous international, national and regional guidelines and strategies developed to address the escalating threat of carbapenem-resistant *Enterobacterales* (CRE) continued to increase worldwide [6, 7]. At present, there was still no consensus on the optimally effective interventions or the best combination of interventions to curtail the spread of CRE. Interventions in healthcare settings most often had involved bundled control measures, which varied by institution and effect [8]. These discrepancies might reflect differences in regional epidemiology that required distinct prevention strategies.

In China, since the first *Klebsiella pneumoniae* (KP) producing KPC-2 was reported in 2007 [9], the prevalence of carbapenem-resistant KP (CRKP), especially the KPC-2 ST11 clones, had increased. Recent data from the CHINET surveillance system showed that imipenem-resistance rate of KP increased from 3.0% in 2005 to 25% in 2018, with resistance rate reaching over 45% in some hospitals [10]. The annual upward trend was also obtained from the China Antimicrobial Resistance Surveillance System (http://www.carss.cn/), which further showed that the annual isolation rate of CRKP in 2014–2019 was for geriatric patients over the age of 65 years 7.4/8.7/9.9/10.2/11.3/12.2 and for adult patients 5.3/6.3/7.4/7.8/8.9/9.7 (%), respectively. In our hospital, the annual isolation rate of CRE increased from 10.2% in 2014 to 15.4% in 2019. However, very few detailed epidemiological data are available to uncover the underlying mechanisms for these increases.

In this context, we sought to determine the genotypic features of CPE in a region and to describe their epidemiological evolution and management by performing a series of integrated analysis of clinical, epidemiologic, microbiologic and molecular data.

## Results

### Genotypic features of CPE isolates

A total of 930 phenotypically confirmed CRE isolates were screened for carbapenemase genes, 922 isolates were detected as CPE positive including 740 isolates in our hospital and 182 isolates in other hospitals. The most frequently isolated CPE strains were KP (*n* = 785), followed by *E. coli* (*n* = 96), *Klebsiella oxytoca* (*n* = 12), *Enterobacter cloacae* (*n* = 9), *Citrobacter freundii* (*n* = 9), *Serratia marcescens* (*n* = 5) and other species (*n* = 6) (Table 1). The most frequently detected carbapenemase genes was *blaKPC-2* (*n* = 780), followed by *blaNDM* (*n* = 127), *blaIMP-4* (n = 7), and *blaKPC-2* + *blaNDM-1/5* genes (*n* = 8) (Table 1).

**Table 1:** Distribution of carbapenemase-producing *Enterobacterales* isolates and their carbapenemase genes

Species	Carbapenemase genes	No. of clinical isolates in our hospital	No. of isolates from rectal and stool in our hospital	No. of clinical isolates in other hospitals
*Klebsiella pneumoniae* (*n* = 785)	*blaKPC-2*	517	90	122
	*blaNDM-1*	11		24
	*blaNDM-5*	6	2	2
	*blaIMP-4*	2		2
	*blaKPC-2* and *blaNDM-1* or *5*	6		1
*Escherichia coli* (*n* = 96)	*blaKPC-2*	30	6	
	*blaNDM-1*	9	1	2
	*blaNDM-5*	23	1	20
	*blaNDM-7*	1		
	*blaNDM-9*	2		1
*Klebsiella oxytoca* (*n* = 12)	*blaKPC-2*	3	1	
	*blaNDM-5*	4	1	
	*blaIMP-4*			3
*Enterobacter cloacae* (*n* = 9)	*blaNDM-1*	7		2
*Citrobacter freundii* (*n* = 9)	*blaKPC-2*	4		
	*blaNDM-1*	4		1
*Serratia marcescens* (*n* = 5)	*blaKPC-2*	4		
	*blaNDM-1*	1		
Other species (*n* = 6)	*blaKPC-2*	2	1	
	*blaNDM-1*			2
	*blaNDM-5* and *blaKPC-2*	1		

MLST and *wzi* assigned 785 carbapenemase-producing KP (CPKP) isolates to 26 STs and 32 *wzi* alleles (*wzis*), respectively. Among them, ST11-*wzi*209 accounted for the largest proportion (56.31%, 442), followed by ST11-*wz*i141 (20.38%, 160), ST15-*wzi*384 (3.82%, 30), ST437-*wzi*109 (3.44%, 27), ST11-*wzi*64 (2.29%, 18) and other STs and *wzi*s (13.76%, 108) (Table 2). The 96 carbapenemase-positive *E. coli* isolates contained 36 KPC-2 isolates belonging to 9 distinct STs with a predominance of ST43 (55.56%, 20/36) and 60 NDM isolates belonging to 20 unique STs (13 isolates unidentified) dominated by ST167 (36.17%, 17/47).

**Table 2:** Distribution of common STs and *wzi* alleles in carbapenemase-producing *Klebsiella pneumoniae*

Sequence types	*wzi* allele**s**	Carbapenemase genes	No. of clinical isolates in our hospital^a^	No. of isolates from rectal and stool in our hospital	No. of clinical isolates in other hospitals^b^
ST11	*wzi209*	*blaKPC-2*	277	58	107
	*wzi141*	*blaKPC-2*	149	8	1
	*wzi141*	*blaKPC-2* and *blaNDM-5*	*2*		
	*wzi64*	*blaKPC-2*	17		1
	*wzi89*	*blaKPC-2*	3	1	1
	*wzi133*	*blaKPC-2*	1		2
	*-*^*a*^	*blaKPC-2*	2	2	2
ST15	*wzi384*	*blaKPC-2*	18	5	7
	*wzi173*	*blaKPC-2*			2
	*wzi19*	*blaKPC-2*	2		1
ST17	*wzi141*	*blaNDM-5*	3	2	
ST20	*wzi84*	*blaNDM-1* or *5*	1		10
ST23	*wzi1*	*blaKPC-2* or *blaNDM-1*	13		
ST437	*wzi109*	*blaKPC-2*	21	6	
ST617	*wzi162*	*blaKPC-2*	6	6	
ST2068	*wzi381*	*blaNDM-1*	1		3
ST307	*wzi173*	*blaIMP-4*			2
ST48	*wzi62*	*blaKPC-2* or *blaNDM-1*	3	1	
ST896	*wzi59*	*blaKPC-2*	3	2	
ST101	*wzi137*	*blaKPC-2*		1	
	*wzi29*	*blaKPC-2*	1		
ST37	*wzi64*	*blaNDM-1*	1		
	*wzi50*	*blaIMP-4* or *blaNDM-1*	1		2
	*wzi14*	*blaNDM-1*			1

a: No detected

^a^No. of clinical isolates in our hospital include: Another 17 isolates belonging to 8 STs and 8 *wzis*

^b^No. of clinical isolates in other hospitals include: Another 9 isolates belonging to 5 STs and 5 *wzis*

In the study, we noticed that there were some differences among CPKP isolates belonging to the same STs and *wzis*, and these differences could be manifested in the colonial morphotypes such as viscosity, size and color, etc. So the morphologically distinct CPKP isolates were further tested for ESBL genes to distinguish clones with the same STs and the same *wzi*s. A total of 296 CPKP isolates were tested for extended spectrum β-lactamase (ESBL) genes, and the results were shown in Table 3. We found that the combinations of different β-lactamase genes could further differentiate some CPKP clones that shared the same STs and the same *wzis* (for example, ST11-*wzi209*). However, the same of some combination of different β-lactamase genes could also be detected among clones belonging to different STs and different *wzi*s.

**Table 3:** Distribution of extended spectrum β-lactamase (ESBL) genes in *Klebsiella pneumoniae* isolates with different STs and different *wzis*

Sequence types	*wzi* allele**s**	Carbapenemase genes	ESBL genes	No.
ST11	*wzi209*	*blaKPC-2*	*blaSHV-11/12/31/155* + *blaTEM-1* + *blaCTM-M-14*	156
			*blaSHV-11* + *blaTEM-1* + *blaCTM-M-15*	2
			*blaTEM-1* + *blaCTX-M-14*	1
			*blaSHV-11/12* + *blaCTX-M-14*	6
			*blaSHV-11/12* + *blaTEM-1*	14
			*blaSHV-1*	2
			*blaSHV-11/12* + *blaTEM-1* + *blaCTX-M-14* + *blaCTX-M-15*	4
	*wzi64*	*blaKPC-2*	*blaSHV-11/12/31* + *blaTEM-1* + *blaCTM-M-14*	9
			*blaSHV-11* + *blaTEM-1* + *blaCTM-M-15*	2
			*blaSHV-11* + *blaCTX-M-15*	2
	*wzi141*	*blaKPC-2*	*blaSHV-12/11* + *blaTEM-1* + *blaCTM-M-14*	28
			*blaSHV-12* + *blaTEM-1* + *blaCTM-M-15*	1
			*blaSHV-12* + *blaTEM-1*	6
			*blaSHV-1*	6
	*wzi89*	*blaKPC-2*	*blaSHV-11* + *blaCTX-M-14*	5
ST15	*wzi384*	*blaKPC-2*	*blaSHV-28* + *blaTEM-1* + *blaCTM-M-14*	14
			*blaTEM-1* + *blaCTX-M-14*	2
			*blaSHV-28* + *blaTEM-1*	2
	*wzi19*	*blaKPC-2*	*blaSHV-28* + *blaCTX-M-14*	1
			*blaSHV-28* + *blaTEM-1* + *blaCTM-M-15*	2
ST23	*wzi1*	*blaKPC-2*	*blaSHV-12* + *blaCTX-M-15*	6
ST437	*wzi109*	*blaKPC-2*	*blaSHV-11* + *blaTEM-1*	19
ST617	*wzi162*	*blaKPC-2*	*blaSHV-12/31* + *blaTEM-1* + *blaCTM-M-15*	6

Among the 533 CRE patients, 528 cases were confirmed to have CPE colonization and/or infection in which 127 consecutive cases with clinical infections underwent rectal and stool screening, 96 of whom had CPE in rectal and stool cultures (75.59%, 96/127). Eighty-six patients infected by CPE strains also intestinally colonized with organisms bearing the same STs. A total of 103 CPE isolates were recovered from the rectal and stool screening cultures. The predominant species identified were KP (*n* = 92), with ST11-*wzi*209 KPC-2 (*n* = 58) KP being the most frequently isolated, followed by *E. coli* (*n* = 8) (Table 1 and 2).

ST11-*wzi*209 KPC-2 KP was established in all hospitals and some of which could be traced back to patients transfers between hospitals. ST20-*wzi*84 NDM-1 KP and ST167 NDM-5 *E. coli* were detected in 5 hospitals. Some ST11 KPC-2 subclones (*wzi*89, *wzi*133, *wzi*64 and *wzi*141) and ST15 KPC-2 subclones (*wzi*173, *wzi*19) based on capsular typing were found in two hospitals, Some STs-*wzi*s or species (ST15-*wzi*384, ST2068-*wzi*381, IMP-4 K*. oxytoca*) were observed in three hospitals, and other relatively rare STs or *wzi*s were only confined to their individual settings.

#### Clinical characteristics of CPE patients

The majority of CPE patients (68%) were male, but no statistical differences in clinical features between sexes were observed (Table 4). Further analysis showed that the mean age of patients with CPE decreased significantly in 2016 compared to 2015 (*P* = 0.034). This could be explained by the rapid increase in the number of patients under the age of 65 years in 2016 (Table 4). One hundred and forty-seven (97.33%, 146/150) of these young cases were nosocomially acquired and the main reasons for primary admission were cerebral hemorrhage (41.45%), motor vehicle accident (11.18%), pulmonary infection (10.53%), fall injury (8.55%) and tumor (6.58%). More than half patients had a previous hospital admission within 1 month before the current admission prior to 2018, but this proportion decreased in 2018. As shown in Table 4, the average length of hospitalization of CPE patients showed a downward trend mainly due to significant increases in voluntarily discharged rates and mortality rates. The number of intra-hospital transfers of CPE patients increased gradually from 18 (17.82%, 101) in 2015 to 63 (30.73%, 205) in 2018 (*p* = 0.016). There was a statistically significant increase in the number of inter-hospital transfers of CPE patients [12 (11.88%, 101) in 2015 vs 51 (24.88%, 205) in 2018 (*p* = 0.008)].

**Table 4:** Characteristics of patients with carbapenemase-producing *Enterobacterales* according to the years

Characteristic	2014–2018		2014 ( *n* = 25) No. (%)	2015 (*n* = 101) No. (%)	2016 (*n* = 104) No. (%)	2017 ( *n* = 90) No. (%)	2018 (*n* = 205) No. (%)
	Male (*n* = 357)	Female (*n* = 168)					
	No. (%)	No. (%)					
Age (years)	72.17 ± 16.55	72.20 ± 15.14	80.36 ± 10.70	76.94 ± 13.60	71.08 ± 17.26	69.84 ± 16.61	70.47 ± 16.27
*P* value	0.641			0.213	0.034	0.494	0.738
Patients under 65 years (No.)	103 (28.85)	47 (27.98)	2 (8)	13 (12.87)	38 (36.54)	34 (37.78)	63 (30.73)
*P* value	0.836			0.743	< 0.001	0.859	0.236
Previous hospitalization within last one month	209 (58.54)	88 (52.38)	19 (76)	71 (70.3)	65 (62.5)	49 (54.44)	93 (45.37)
*P* value	0.184			0.572	0.238	0.256	0.151
Hospital length of stay (days)	42.20 ± 37.56	42.35 ± 41.08	53.96 ± 37.69	50.11 ± 41.64	45.05 ± 44.62	41.99 ± 33.05	34.19 ± 28.61
*P* value	0.882			0.748	0.093	0.793	0.069
Outcome							
Improvement	191 (53.5)	92 (54.76)	8 (33.33)	52 (54.74)	68 (65.38)	59 (65.56)	96 (46.83)
Voluntary discharge	61 (17.09)	36 (21.43)	3 (12.5)	17 (17.89)	15 (14.42)	15 (16.67)	47 (22.93)
Death	82 (22.97)	32 (19.05)	13 (54.17)	26 (27.37)	20 (19.23)	13 (14.44)	42 (20.49)
*P* value	0.375			0.051	0.256	0.677	0.047
Intra-hospital transfers	105 (29.41)	36 (21.43)	8 (32)	18 (17.82)	27 (25.96)	25 (27.78)	63 (30.73)
*P* value	0.054			0.117	0.159	0.776	0.61 (0.016*)
Inter-hospital transfers	74 (20.73)	24 (14.29)	7 (28)	12 (11.88)	13 (12.5)	15 (16.67)	51 (24.88)
*P* value	0.077			0.088	0.892	0.41	0.119 ( 0.008*)

*P* values were comparisons between sexes or comparisons between adjacent two year

^*^P value was the comparison between 2018 and 2015

#### Epidemiological evolution of ST11 KP clones

**Fig. 1:**
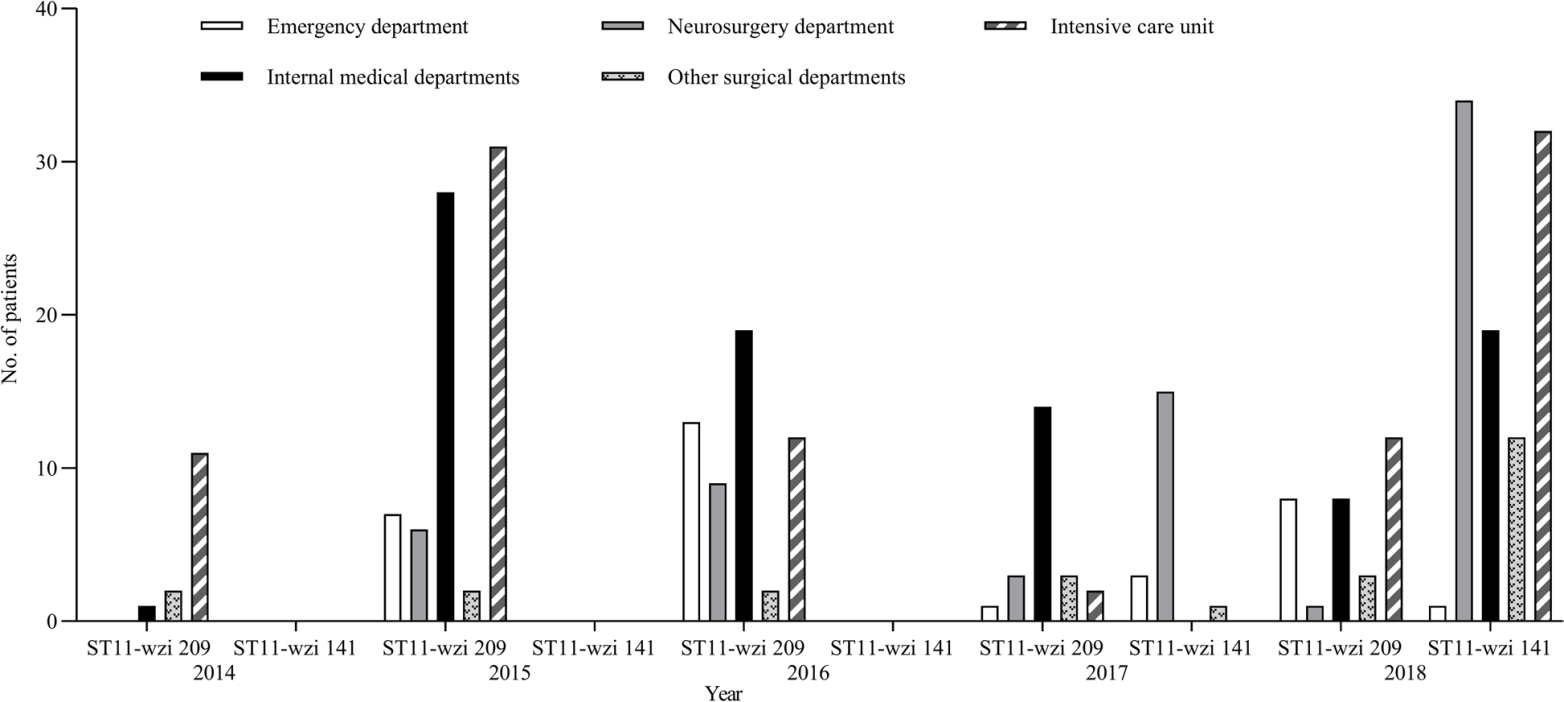
Distribution of patients with KPC-2 producing *Klebsiella pneumoniae* of ST11-*wzi*209 or ST11-*wzi*141 in our hospital according to the years and departments

**Fig. 2:**
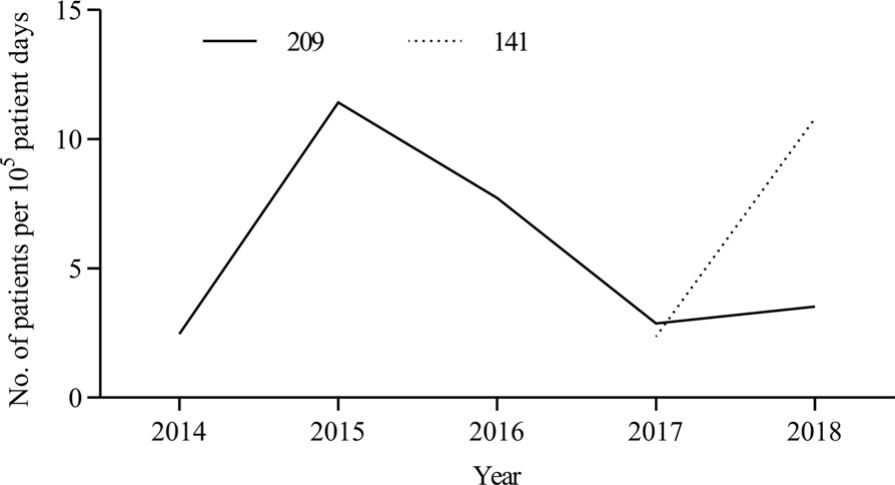
Incidence of patients with KPC-2 producing *Klebsiella pneumoniae* of ST11-*wzi*209 or ST11-*wzi*141 per 10^5^ patient days according to the years

The ST11-*wzi*209 clone was first detected in February 2014 in a patient transferred to the ICU in our hospital, and its number had increased since August 2014. The clone caused an outbreak involving 12 patients in the ICU during March 15-April 2015. A multidisciplinary infection control team was then formed in the ICU in May 2015 to identify problems regarding nosocomial infection control and develop practical way to limit the transmission of the clone. Some basic control measures were enhanced immediately, including twice-weekly training of medical staff regarding CRE, with special emphasis on hand hygiene and contact precautions, partial restrictions on ward admission, and extensive environmental screenings for room surfaces, equipment, and staff hands. In multiple environmental samples, only one isolate of ST11-*wzi*209 was recovered from a bed sheet after ultraviolet light disinfection, suggesting an important model of transmission via the hands of healthcare workers from the contaminated bed linens to new patients and the inadequate disinfection. Evidence-based control measures were then developed and gradually implemented in the ICU since July: patient wiping with chlorhexidine once per day, disinfection of bed linens with an ozone sterilizer twice a week, reinforcement of patients’ environmental disinfection with chlorine-based compound twice-daily, and inspection of strict adherence to hand hygiene and compliance with contact precautions. Afterward, a substantial decrease was observed in the number of new cases in the ICU (Fig. 1). In March 2016, considering the rapid increase of CRE in our hospital and the continuing risk of importation pressures, the above measures were gradually taken in most wards of our hospitals, and we observed a significant drop in the incidence of ST11-*wzi*209 KP acquisition (Fig. 2). Notably, the number of new patients who were colonized and infected with ST11-*wzi*209 KP in the ICU and the emergency wards increased slightly in 2018, suggesting that infection control measures still needed to be further improved. However, stringent implementation of these measures had been maintained to avoid further transmission of the clone in our hospital.

The ST11-*wzi*141 clone initially appeared in neurosurgery ward in April 2017 and spread rapidly to different wards, causing several outbreaks in the ICU and neurosurgery wards largely due to frequent patient transfers between both wards. Successive attempts failed to identify sources or reservoirs of the epidemic clone during the surveillance of the affected wards’ environment. Despite the implementation of the above measures that had greatly reduced the transmission of the ST11-*wzi*209 clone in our hospital, the widespread transmission of the ST11-*wzi*141clone to various wards were not contained, and it had evolved into an endemic situation in the hospital (Fig. 1).

The ST11-*wzi*64 clone was introduced into the emergency department in December 2013, resulting in 3 cases of infection. The clone was detected in 2 cases in 2014, 3 cases in 2015 and 1 case in 2016 in the emergency department of our hospital, but not in other wards. None cases with the clone were found in 2017. However, this clone re-emerged in 2018, with 7 new cases in five different wards. The ST11-*wzi*89 clone and ST11-*wzi*133 clone were found in the Department of Infectious Diseases in March 2016 and the ICU in May 2018, respectively. No signs of their transmission in the hospital were found and no special control measures were taken.

#### Epidemiological features of carbapenemase-producing *E. Coli*

**Fig. 3:**
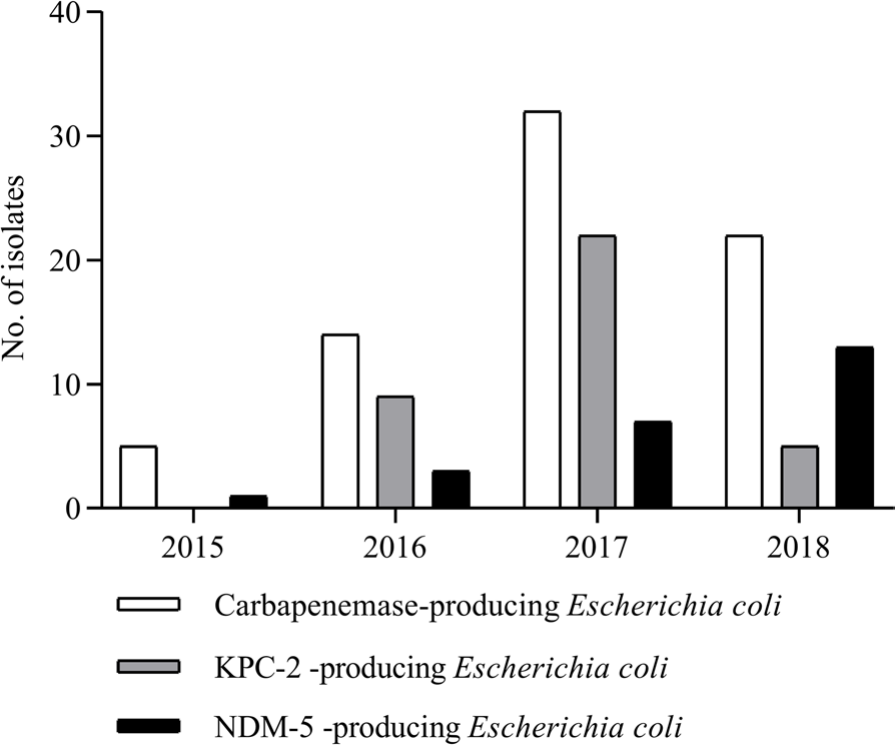
Distribution of carbapenemase-producing *Escherichia coli* isolates in our hospital according to the years

A total of 73 carbapenemase-producing *E.coli* isolates were recovered from 64 patients in our hospital during the study period, including 36 isolates harboring *blaKPC-2* recovered from 30 patients and 37 isolates with *blaNDM* from 34 patients, of which 5 patients with NDM *E.coli* were transferred from other hospitals and the remaining 59 cases were all hospital acquired. The KPC-2 *E.coli* was first detected in our hospital in June 2016, but not in other hospitals. Due to an outbreak of ST43 *E.coli* in neurosurgery ward, its number increased markedly in 2017 (Fig. 3). This clone first appeared in January 2017, lasted for 6 months and disappeared since July 2017. A total of 16 patients acquired pneumonia infection with this clone and 3 cases died. However, the clone was not detected in other wards in our hospital. The first NDM *E.coli* appeared in January 2015, and the number gradually increased, especially NDM-5 isolates (Fig. 3), which were distributed in different wards and had not caused any substantial outbreaks. These NDM-5 belonged to a variety of STs, with ST167 being more common.

#### Correlation between antibiotic consumption and CRE prevalence

Yearly consumption rate of carbapenems decreased from 38.95 in 2016 to 27.22 DDDs/1000 PDs in 2018, while the usage of third-generation cephalosporins increased from 37.98 in 2016 to 68.39 DDDs/1000 PDs in 2018 in our hospital. No significant associations of annual CRE prevalence were found with yearly consumption rates of carbapenems (*r* = -0.13, *P* = 0.806), fluoroquinolones (*r* = -0.301, *P* = 0.562), first-generation cephalosporins (*r* = 0.732, *P* = 0.098), second-generation cephalosporins (*r* = -0.529, *P* = 0.280), third-generation cephalosporins (*r* = -0.1, *P* = 0.851), and beta-lactam/beta-lactamase inhibitor combinations (*r* = -0.485, *P* = 0.329).

#### Transferability of carbapenemase genes via plasmid conjugation

A total of 210 clinical CPE isolates were tested for mobility of carbapenemase-bearing plasmids by conjugation with *E. coli* J53, 49 isolates were successfully transferred. High conjugation rates were found among NDM plasmids in both CPKP (54.84%, 17/31) and *E. coli* (46.67%, 14/30). The conjugation rates of the KPC-2 plasmids in *E.coli* and CPKP were 25% (2/8) and 10.08% (12/119), respectively. Nine isolates of *Klebsiella oxytoca* that included 3 KPC-2 isolates, 3 NDM-1 isolates and 3 IMP-4 isolates were conjugated with *E. coli* J53, only isolates that producing KPC-2 achieved conjugative transfer. All ST15-*wzi*19 KPC-2-producing KP isolates were readily transferable whereas all ST15-*wzi*384 KPC-2 KP were not conjugative. Among the ST11 KPC-2 KP clones, isolates of ST11-*wzi*209 (*n* = 53), ST11-*wzi*64 (*n* = 5), ST11-*wzi*89 (*n* = 3) and ST11-*wzi*133 (*n* = 2) failed to transfer, while only one strain of the 7 ST11-*wzi*141 isolates was able to transfer its plasmid, suggesting that different STs and different *wzi*s could influence the conjugation efficiency.

The results of in vitro reverse conjugation experiments showed that the *blaKPC-2*-bearing plasmid derived from KPC-2-producing *K. oxytoca* strain had a weaker conjugal transferability than the *blaKPC-2*-bearing plasmid derived from ST15-*wzi*19 KPC-2-producing KP strain, suggesting that transconjugants from different origins could have different conjugal transferabilities (Table 5). Among the 31 carbapenem-susceptible KP (CS-KP) isolates receiving *blaKPC-2*-bearing plasmid, some isolates could accept plasmids at 25 °C, some could at 37 °C, and some could at both temperatures, suggesting that different STs, different *wzi*s and temperature could influence plasmid conjugation efficiency (Table 5).

**Table 5:** Transferability of carbapenemase genes via in vitro reverse conjugation experiment

Specimens	Carbapenem-susceptible KP		*E. coli* J53 T _kI_		*E. coli* J53 T _kII_	
	*wzi* type	ST type	25 °C	37 °C	25 °C	37 °C
Sputum	89	11	+	+	-	+
Sputum	64	11	+	-	-	-
Sputum	\	11	+	+	+	-
Stool	1	23	-	+	-	-
Sputum	1	23	+	+	+	+
Environment	29	101	+	+	+	+
Stool	29	101	-	-	-	-
Sputum	29	101	+	+	-	-
Stool	50	873	-	-	-	-
Stool	50	1073	+	+	-	-
Pus	173	1505	+	+	-	-
Stool	173	307	-	-	-	-
Blood	84	20	-	-	-	-
Pus	114	34	+	+	+	+
Sputum	27	36	+	+	-	-
Sputum	438	37	+	+	+	+
Stool	83	39	-	-	-	-
Sputum	101	45	-	-	-	-
Sputum	377	107	+	-	+	-
Sputum	47	133	+	+	-	-
Stool	39	200	-	-	-	-
Sputum	417	279	-	-	-	-
Sputum	42	309	+	-	+	-
Sputum	206	412	+	+	+	+
Stool	80	469	-	-	-	-
Sputum	69	681	+	-	-	-
Stool	59	896	+	+	-	-
Stool	25	1565	-	-	-	-
Sputum	60	1224	-	+	+	+
Blood	446	1847	-	-	-	-
Pus	193	2459	+	+	+	-

\: No detected; + : Successfully transferred; -: No transferred

## Discussion

In this study, a vast majority of the CPE isolates collected from 19 hospitals in our region were CPKP, with ST11 KPC-2 KP being the most common isolates. Different combinations of extended spectrum-β-lactamase genes of *blaSHV*, *blaCTX*, and *blaTEM* were found in KPC-2 producing KP isolates. The *wzi* typing method could further divide CPKP clone of the same ST into different subclones. Conjugation assays showed that horizontal gene transfer events were very low for the most common KPC-2 KP strains, suggesting that the clonal spread of CPKP was the main factor contributing to the CPE increase in the region.

Various strains of KPC-producing KP had disseminated globally and caused numerous outbreaks in many healthcare settings, which posed an immense challenge to infection control due to their high ability to spread and rapid acquisition of drug resistant traits [11]. So accurate strain typing is especially necessary to recognize endemic clonal groups and track their transmission worldwide. Pulsed field gel electrophoresis (PFGE) were the most important molecular typing tools for the analysis of bacterial strain clonality. However, the method was associated with poor reproducibility and often tended to produce subjective biases, making direct comparison of data across different laboratories an immense challenge [12, 13]. Moreover, it was not suitable for precisely tracking CRKP epidemics due to their high clonality [14]. D'Andrea et al. [11] found that ST258 or ST512, the most successful global spread of KPC-KP, had diverse capsular polysaccharide (CPS) genes, and that there were significant differences in the transmissibility between clones with different CPS genes. A similar study conducted at a tertiary care hospital in New York City showed that six *wzi* allele types were detected among 32 ST258 isolates, Within ST258, clones carrying the *wzi*154 allele were associated with higher mortality rates and were also responsible for local expansion or interhospital spread [15]. However, detailed knowledge on the CPS genes of KPC-2 KP in China is still limited. In this study, we observed at least five *wzi* alleles among the epidemic ST11 KPC-2 KP. The clones carrying *wzi*209 and *wzi*141 were the most frequently encountered isolates with high potential for rapid transmission, with the former spreading throughout the region and the latter disseminating in most departments of our hospital. However, the clones carrying *wzi*64 and *wzi*89 were only restricted to the emergency department and the infectious disease department, respectively. So monitoring the trends and extent of transmission of these *wzi* alleles in the region could help inform the design of targeted interventions to limit their transmission.

Capsular polysaccharide was considered to be a virulence factor for KP, and some capsular types such as K1, K2, K5, K20, K54 and K57 were associated with invasive diseases or unique pathogenicity [16–18]. CPKP ST11 was increasingly becoming hypervirulent especially in China and two CPS (K64 and K47) in ST11 isolates were found to be carbapenem-resistant hypervirulent KP [16, 17]. The hypervirulence status of the most prevalent CPKP isolates such as *wzi*209 and *wzi*141 in our study was also determined by polymerase chain reaction (PCR) amplification of virulence plasmid associated genes (*rmpA2*, *iutA*, *iucA*) [18], which were identified as hypervirulent KP, but none of the isolates tested in our study carried these virulence genes.

The presence of ESBLs, AmpC β-lactamases and carbapenemases in *Enterobacterales* isolates conferred resistance to both β-lactam antibiotics and some non-β-lactam antibiotics, which posed a major challenge to treatment [19]. In China, the prevalence of ESBLs in healthcare settings and community settings was rapidly increasing, with *blaCTX-M-15* and *blaCTX-M-14* being the predominant ESBL genotypes [20]. It was noteworthy that ESBL genes such as *blaCTX-M*, *blaSHV* and *blaTEM* were also detected in KPC-producing KP isolates [21, 22]. In the study, the combinations of different ESBL genes were identified in our KPC-2-producing KP isolates, with *blaCTM-M-14/15*, *blaSHV-11/12* and *blaTEM-1* being the common ESBL genotypes.

The rapid increase in the prevalence of CRE in China was due to both horizontal dissemination of resistance genes between bacteria as well as clonal expansion and transmission [23, 24]. The *blaKPC-2* gene could be transferred between different species of *Enterobacteriaceae* family through conjugative plasmids, and the transferability of *blaKPC-2*-carrying plasmid was not restricted to a certain transposon, ST or PFGE subtype [21]. Our conjugation results showed that the carbapenemase genes could reside on transferable plasmids, but plasmid transfer was very low for the most prevalent types of KP strains, namely ST11-*wzi*209 and ST11-*wzi*141, suggesting that plasmid transfer was not the major driver of CPE transmission of these strains in our region. However, novel CRKP strains with different STs and/or *wzi*s and new carbapenemase-producing *E.coli* with different STs were frequently detected during the study period, suggesting unconstrained plasmid transfer. Of great concern was the emergence of strains that co-producing KPC-2 and NDM-1 or 5, especially KP ST11-*wzi*141 that co-producing KPC-2 and NDM-5, which would further limit clinicians’ choice of antibiotics for these infections. Therefore, further exploring the underlying mechanisms influencing horizontal plasmid transfer might help design effective ways to block them. Factors such as temperature, substrate, plasmid content, and donor and recipient strain identity influenced conjugation rate [25]. In this study, conjugation efficiency was influenced by different STs, different *wzi*s and temperature.

Unnecessary and excessive use of antibiotics usually led to the emergence, colonization, clonal expansion and plasmid transmission of CRE strains [26, 27]. There exsisted a close correlation between the carbapenem consumption and the rate of carbapenem-resistant gram negative bacilli [28]. However, there were studies suggesting no effect of change in antimicrobial use in reducing CRKP infections [29, 30]. The current work did not find any association between antimicrobial consumption and CRE prevalence, the possible reason might be that the rapid spread of resistant bacteria and subsequent outbreaks masked these relationships.

Although basic control measures had been strictly implemented throughout the study period, they were fundamentally inadequate to curb CPKP transmission.The implementation of evidence-based control measures greatly reduced the spread of ST11-*wzi*209 clone in our hospital during the study period, but insufficient to control the transmission of ST11-*wzi*141 clone after its introduction to the hospital. This clone had spread to all wards and become the main clone, and it still continued to accrue despite these control measures taken, suggesting a higher transmission capacity or greater epidemic potential than ST11-*wzi*209 clone. The same Intervention(s) might have different effects on different bacteria due to different modes of transmission [31–33]. Our findings demonstrated that different clones had different transmission features and required different preventative strategies. The lack of success in the control of ST11-*wzi*141 clone indicated that other determinants that contributed to the sustained transmission were still needed to be explored in further studies. Despite showing great promise in reducing the transmission of ST11-*wzi*209 KP following the implementation of these control measures in ICU and emergency wards, the number of new cases increased in 2018, suggesting that some problems that might undermine control efforts were desperately needed to be addressed: the lack of routine CRE rectal surveillance program for high-risk patients, no obvious environmental sources despite repeated environmental samplings, overcrowding and medical staff shortages. Considering the increasing transferred patients from other wards or other hospitals, and the large number of immunocompromised patients in these wards, active surveillance with admission screening in these settings should be particularly appreciated and initiated.

Gastrointestinal colonization of CRE in patients was an important risk factor for the dissemination of these bacteria in the healthcare settings, and active rectal surveillance cultures to detect asymptomatic colonization in patients were very essential for the development effective control measures [34, 35]. Numerous studies had demonstrated the effectiveness of active surveillance in controlling the transmission and outbreaks of CRE [29, 33, 36]. The rectal CPE carriage rates varied greatly from different geographic areas of China. Zhao et al. [37] reported that a prevalence of rectal carriage of CPE in patients was 2.6%, while Liu et al. [38] showed that the fecal carriage rate of CPE was 7.81% in their patients. However, these studies failed to address which types of clinical infectious CPE were more likely to colonize the gastrointestinal tract. In the study, we found that some ST11 KP epidemic clones such as *wzi*209 and *wzi*141 could colonize the intestinal tract. Although the ST11-*wzi*89 KP clone had been detected in the intestinal tract, it had a more restricted transmission. Other strains with high transmission potential, such as ST15-*wzi*384 KPC-2 KP, ST437-*wzi*109 KPC-2 KP and ST43 KPC-2 *E.coli*, were also found to colonize the intestinal tract. Therefore, exploring the intestinal colonization profiles of clinical CPE isolates could highlight the necessity of active rectal surveillance, and also facilitate early initiation of prevention and control measures that targeted different CPE strains.

The spread of CRE across hospitals due to the mobility of patients was increasingly recognized as an important mode of transmission [39–41]. Our findings indicated that transfer events of CPE patients between hospitals presented a rising trend, suggesting that most hospitals would experience an increasing frequency of CPE introduction without any inter-hospital CPE status communication. In China, patients usually chose hospitals completely according to their individual preference without any restriction from regional or local health authorities. This situation might provide great opportunities for the movement of asymptomatic carriers to transmit CPE. Currently, almost all infection control efforts focused exclusively on individual institutions, while regional or national guidelines for controlling the dissemination of CRE across health care settings were still deficient. The values of regional/national strategies had been demonstrated by experiences in Israel [34] and others [42, 43], where CRE prevalence could be effectively controlled through coordinated interventions. Our findings indicated that active rectal screening cultures, development of various adequate CPE molecular typing in clinical laboratories, especially *wzi* typing, timely communication of CPE status among institutions or wards upon patient transfer, and public education regarding CPE transmission routes and effective preventive measures would be essential control measures to address the future CPE challenge.

## Conclusions

CPE were still evolving and spreading rapidly, and they would become a persistent problem in the future, mainly due to the continuing risk of importation pressures and deficits in regional strategies. Hence, continued vigilance for the importations should be maintained, and comprehensive epidemiological studies specific for institutions or regions were urgently needed to develop effective control measures to combat the ever-changing threat.

## Methods

### Samplings

This study was conducted from December 1, 2013 to December 31, 2018 in Hebei General Hospital, a 1830-bed tertiary care hospital located in Hebei, China. Seven secondary hospitals and 11 other tertiary-care hospitals located in geographically separated areas in this province voluntarily participated in the study. The other 18 hospitals were requested to collect CRE isolates from clinical samples as well as the related basic clinical and epidemiological data between September 2015 and December 2017. For strains that caused outbreaks in these hospitals, only one representative strain from each sample was collected. During the study period, a total of 533 patients in our hospital were confirmed to be CRE patients, of which 643 CRE isolates were from various clinical diagnostic specimens and 103 CRE isolates were from rectal and stool. More than one colony have been isolated in our samples.

Another 184 CRE isolates were collected from other 18 hospitals. The species and specimen distribution of 930 CRE strains including 746 isolates from our hospital and 184 isolates from other hospitals were shown in Table 6. CRE/CPE cases were defined by the isolation of CRE/CPE in any biological sample obtained from the patients. Nosocomial acquisition was defined as positive culture results obtained > 48 h after hospital admission or ≤ 48 h if the patient had a known history of CRE acquisition in the previous year. The demographic, clinical and epidemiological data were collected via electronic medical record review. Infection and/or colonization were defined according to the criteria established by the U.S. Centers for Disease Control and Prevention (CDC) [44].

**Table 6:** Species and specimen distribution of 930 carbapenem resistant *Enterobacteriaceae* strains including 746 isolates from 533 patients in our hospital and 184 isolates from 184 patients in other hospitals

Species	Speccimen type [n (%)]^a^									
	Respiratory	Urine	Rectal and stool	Blood	Abscess & Wound	Drainage fluid	Central vein catheter	Cerebrospinal fluid	Bile	Total
*Klebsiella pneumoniae*	517 (65.61)	76 (9.64)	92 (11.68)	42 (5.33)	17 (2.16)	23 (2.92)	8 (1.02)	8 (1.02)	5 (0.63)	788
*Escherichia coli*	43 (43.43)	26 (26.26)	8 (8.08)	8(8.08)	9 (9.09)	1 (1.01)	1 (1.01)		3 (3.03)	99
*Klebsiella oxytoca*	5	4	2	1						12
*Enterobacter cloacae*	6	3								9
*Citrobacter freundii*	4	3		2						9
*Serratia marcescens*	4			1						5
*Klebsiella aerogenes*		1		1						2
Other species	3	1	1		1					6
Total	582 (62.58)	114 (12.26)	103 (11.08)	55 (5.91)	27 (2.90)	24 (2.58)	9 (0.97)	8 (0.86)	8 (0.86)	930

^a^calculated for *Klebsiella pneumoniae* and *Escherichia coli* only

### Microbial identification and antibiotic susceptibility testing

Various clinically indicated samples were routinely streaked onto blood agar plates and eosin methylene blue agar (EMB) plates (blood and other body fluids must do enrichment culture) and incubated aerobically for 48 h at 35 °C. Screening samples (rectal and stool) and environmental samples were inoculated directly onto EMB plates containing meropenem (1 mg/L) and incubated at 35 °C for 48 h. The suspected colonies grown on the selective medium after incubation were subcultured onto Mueller–Hinton agar on which 10 μg meropenem disk and 10 μg imipenem disk were placed which allowed the establishment of optimal zone diameters for the screening of CRE. After incubation for 24 h at 35 °C, a reduced zone of inhibition of ≤ 22 mm around the meropenem and/or imipenem disc was considered a potential CRE. Bacterial isolates were identified to the species level by MALDI-TOF MS. The susceptibility of bacterial isolates to different antimicrobial agents were determined using the VITEK2 system (bioMe´rieux) and the AST-GN09 card following the manufacturer’s instructions and interpreted according to the criteria of the latest Clinical Laboratory Standards Institute (CLSI) document. Meropenem and imipenem susceptibilities were also carried out by the Kirby-Bauer disk diffusion method on Mueller–Hinton agar for confirmation if a discrepancy in susceptibilities between carbapenems by Vitek 2 occurred. Enterobacterial isolates resistant to at least one of the carbapenems or positive for carbapenemase genes were regarded as CRE. ESBL phenotype was carried out by the standard double disc synergy test with cefotaxime (30 ug) and ceftazidime (30 ug) alone and in combination with clavulanic acid (10 ug) [20]. *E. coli* ATCC 25,922 was used as a quality control.

### Detection of carbapenemase genes and bacterial typing

Resistance genes of non-repetitive CRE isolates were tested by PCR, which included carbapenemase genes of the family of class A (*blaKPC*, *blaNMC*, *blaGES*, *blaIMI* and *blaSME*), class B (*blaVIM*, *blaIMP*, *blaNDM*, *blaSIM*, *blaSPM* and *blaGIM*) and class D (*blaOXA-48*, *blaOXA-23*, *blaOXA-24*, *blaOXA-51* and *blaOXA-58*), and ESBL genes (*blaTEM*, *blaSHV* and *blaCTX-M*) [45, 46], and the PCR products were sequenced and compared with the reported sequences from GenBank by Blast. Bacterial typing was performed by multilocus sequence typing (MLST) using primers listed in the online databases ( https://bigsdb.pasteur.fr/klebsiella/klebsiella.html for KP and https://bigsdb.pasteur.fr/ecoli/primers_used.html for E. coli) and by *wzi* typing for KP isolates [47]. The sequences of amplicons were compared with the existing alleles available from the *wzi* and MLST database (https://bigsdb.pasteur.fr/), and the allelic number was determined for each sequence.

### Intervention measures

Evidence-based guidelines on prevention and control of CRE were unavailable in China. Infection control measures for CRE employed in all participating hospitals were based primarily on the guidelines for the control of multi-drug resistant (MDR) organisms and typically included hand hygiene, contact precautions and/or isolation precautions, healthcare staff education, environmental cleaning and disinfection, aseptic procedures and antimicrobial stewardship (AMS) policies.

In our institution, infection surveillance and control were conducted by the department of preventive medicine. Microbiology laboratory, clinical departments and preventive medicine department shared daily data about MDR organisms. The basic control measures for CRE prevention were as follows: hand hygiene with alcohol-based hand rubs, patients with CRE isolates placed on contact precautions, CRE status marked in the electronic medical record and environment disinfection with a chlorine-based compound. Monthly environmental surveillance cultures were performed for relevant wards, which included air sedimentation cultures, hand swab cultures and environmental swab cultures. Since the release of the National Action Plan to Contain Antimicrobial Resistance (2016–2020) in September 2016, antimicrobial stewardship had been reinforced. Carbapenems were added in the restricted antibiotic list, requiring preauthorization of prescriptions. Monthly multidisciplinary antibiotic meeting was undertaken in the whole hospital, with a particular emphasis on rational use of antibiotics.

When an outbreak occurred, an infection control task force was established immediately and multidisciplinary meetings were frequently held to explore additional effective control measures. Investigative findings on the basis of clinical observations, epidemiological surveys and molecular evidence were immediately fed back to guide development or refinement of control measures.

### Antibiotic consumption

Data on the antibiotic utilization information for all inpatients in our hospital were obtained from hospital pharmacy database. The rates of antibiotic consumption were expressed as the defined daily dose (DDD)/1000 patient-days (PDs). Four main classes of antibacterial agents including carbapenems, fluoroquinolones, cephalosporins, and beta-lactam/beta-lactamase inhibitor combinations were analysed in this study.

### Conjugation experiments

To determine the transferability of the carbapenemase genes, a conjugation experiment was carried out in mixed broth cultures at 35 °C [48]. Carbapenemase positive donor strains from various clinical samples were randomly selected according to the colonial morphotypes (size, color, viscosity, etc.). Azide-resistant *E. coli* J53 was used as recipient. To determine possible factors influencing the conjugation efficiency, an in vitro reverse conjugation experiment was attempted at both 25 °C and 37 °C. A total of 31 CS-KP isolates collected from patients and environment belonging to 26 different STs and 25 different *wzi*s, including 5 isolates with the same STs and the same *wzis* but from different specimens, were selected as recipients. These CS-KP isolates were also susceptive to cephalosporins. Two *E. coli* J53 KPC-2-producing transconjugants (T _kI_ and T _kII_) separately derived from ST15-*wzi*19 KPC-2 producing KP (designated as k I) and KPC-2 producing *Klebsiella oxytoca* (kII) during the conjugation experiments were used as the donors. Transconjugants were initially selected on EMB containing azide (200 μg/ml) and meropenem (1 mg/L) according to the colonial morphotypes. Identification and inspection were performed by MALDI-TOF MS and PCR, respectively.

### Statistical analysis

Data were reported as mean ± SD, number, percentage or frequency according to data distribution. Qualitative variables were compared by Student’s t tests or Mann–Whitney U tests, while categorical variables were compared by Chi-squared or Fisher’s exact tests. Pearson’s or Spearman’s correlation coefficient was used to determine the associations between variables. All tests were two-tailed and *P *values of < 0.05 were considered statistically significant. All analyses were conducted using SPSS software.

## Data Availability

The datasets used and analysed during the current study are available from the corresponding author on reasonable request.

## Abbreviations

CRE: Carbapenem-resistant *Enterobacterales*
CPE: Carbapenemase-producing *Enterobacterales*
KP: *Klebsiella pneumoniae*
CRKP: Carbapenem-resistant *Klebsiella pneumoniae*
CPKP: Carbapenemase-producing *Klebsiella pneumoniae*
MDR: Multi-drug resistant
CS-KP: Carbapenem-susceptible KP
ESBL: Extended spectrum β-lactamase
*wzi*s: *wzi* alleles
ST: Sequence type
CPS: Capsular polysaccharide

## References

[1] Lord Soulsby of Swaffham Prior (2008). The 2008 Garrod lecture: antimicrobial resistance-animals and the environment. J Antimicrob Chemother.

[2] Gupta N, Limbago BM, Patel JB, Kallen AJ (2011). Carbapenem-resistant enterobacteriaceae: epidemiology and prevention. Clin Infect Dis.

[3] van Duin D, Cober ED, Richter SS, Perez F, Cline M, Kaye KS (2014). Tigecycline therapy for carbapenem-resistant Klebsiella pneumoniae (CRKP) bacteriuria leads to tigecycline resistance. Clin Microbiol Infect.

[4] Beyrouthy R, Robin F, Lessene A, Lacombat I, Dortet L, Naas T (2017). MCR-1 and OXA-48 in vivo acquisition in KPC-Producing escherichia coli after colistin treatment. Antimicrob Agents Chemother.

[5] Shields RK, Potoski BA, Haidar G, Hao B, Doi Y, Chen L (2016). Clinical outcomes, drug toxicity, and emergence of ceftazidime-avibactam resistance among patients treated for carbapenem-resistant enterobacteriaceae infections. Clin Infect Dis.

[6] Temkin E, Adler A, Lerner A, Carmeli Y (2014). Carbapenem-resistant enterobacteriaceae: biology, epidemiology, and management. Ann N Y Acad Sci.

[7] Tzouvelekis LS, Markogiannakis A, Psichogiou M, Tassios PT, Daikos GL (2012). Carbapenemases in Klebsiella pneumoniae and other enterobacteriaceae: an evolving crisis of global dimensions. Clin Microbiol Rev.

[8] Munoz-Price LS, Quinn JP (2013). Deconstructing the infection control bundles for the containment of carbapenem-resistant enterobacteriaceae. Curr Opin Infect Dis.

[9] Wei ZQ, Du XX, Yu YS, Shen P, Chen YG, Li LJ (2007). Plasmid-mediated KPC-2 in a Klebsiella pneumoniae isolate from China. Antimicrob Agents Chemother.

[10] Hu F, Guo Y, Yang Y, Zheng Y, Wu S, Jiang X (2019). Resistance reported from China antimicrobial surveillance network (CHINET) in 2018. Eur J Clin Microbiol Infect Dis.

[11] D’Andrea MM, Amisano F, Giani T, Conte V, Ciacci N, Ambretti S, et al. Diversity of capsular polysaccharide gene clusters in Kpc-producing Klebsiella pneumoniae clinical isolates of sequence type 258 involved in the Italian epidemic. PloS One. 2014;9(5):e96827. 10.1371/journal.pone.0096827 PMC4019520 24823690

[12] Bartual SG, Seifert H, Hippler C, Luzon MA, Wisplinghoff H, Rodríguez-Valera F (2005). Development of a multilocus sequence typing scheme for characterization of clinical isolates of Acinetobacter baumannii. J Clin Microbiol.

[13] Sekse C, Sunde M, Lindstedt BA, Hopp P, Bruheim T, Cudjoe KS (2011). Potentially human-pathogenic Escherichia coli O26 in Norwegian sheepflocks. Appl Environ Microbiol.

[14] Snitkin ES, Zelazny AM, Thomas PJ, Stock F, Henderson DK, NISC Comparative Sequencing Program Group (2012). Tracking a hospital outbreak of carbapenem-resistant Klebsiella pneumoniae with whole-genome sequencing. Sci Transl Med.

[15] Gomez-Simmonds A, Greenman M, Sullivan SB, Tanner JP, Sowash MG, Whittier S (2015). Population structure of Klebsiella pneumoniae causing bloodstream infections at a New York City tertiary care hospital: diversification of multidrug-resistant isolates. J Clin Microbiol.

[16] Zhang Y, Jin L, Ouyang P, Wang Q, Wang R, Wang J (2020). Evolution of hypervirulence in carbapenem-resistant Klebsiella pneumoniae in China: a multicentre, molecular epidemiological analysis. J Antimicrob Chemother.

[17] Zhou C, Wu Q, He L, Zhang H, Xu M, Yuan B (2021). Clinical and molecular characteristics of carbapenem-resistant hypervirulent klebsiella pneumoniae isolates in a tertiary hospital in Shanghai. China Infect Drug Resist.

[18] Li J, Ren J, Wang W, Wang G, Gu G, Wu X (2018). Risk factors and clinical outcomes of hypervirulent Klebsiella pneumoniae induced bloodstream infections. Eur J Clin Microbiol Infect Dis.

[19] Rodríguez-Baño J, Gutiérrez-Gutiérrez B, Machuca I, Pascual A (2018). Treatment of Infections caused by extended-spectrum-beta-lactamase-, AmpC-, and carbapenemase-producing enterobacteriaceae. Clin Microbiol Rev.

[20] Zhang J, Zhou K, Zheng B, Zhao L, Shen P, Ji J (2016). High prevalence of ESBL-producing klebsiella pneumoniae causing community-onset infections in China. Front Microbiol.

[21] Liu H, Lin H, Sun Z, Zhu X, Zhang X, Li Q (2021). Distribution of β-lactamase genes and genetic context of bla KPC-2 in clinical carbapenemase-producing klebsiella pneumoniae Isolates. Infect Drug Resist.

[22] Zhang WX, Chen HY, Chen C, Chen JH, Wan FS, Li LX (2021). Resistance phenotype and molecular epidemiology of carbapenem-resistant klebsiella pneumoniae isolates in Shanghai. Microb Drug Resist.

[23] Zhang R, Liu L, Zhou H, Chan EW, Li J, Fang Y (2017). Nationwide surveillance of clinical carbapenem-resistant enterobacteriaceae (CRE) strains in China. EBioMedicine.

[24] Wang X, Chen G, Wu X, Wang L, Cai J, Chan EW (2015). Increased prevalence of carbapenem resistant Enterobacteriaceae in hospital setting due to cross-species transmission of the bla NDM-1 element and clonal spread of progenitor resistant strains. Front Microbiol.

[25] Hardiman CA, Weingarten RA, Conlan S, Khil P, Dekker JP, Mathers AJ (2016). Horizontal transfer of carbapenemase-encoding plasmids and comparison with hospital epidemiology data. Antimicrob Agents Chemother.

[26] Rooney CM, Sheppard AE, Clark E, Davies K, Hubbard ATM, Sebra R (2019). Dissemination of multiple carbapenem resistance genes in an in vitro gut model simulating the human colon. J Antimicrob Chemother.

[27] Ye L, Chan EWC, Chen S (2019). Selective and suppressive effects of antibiotics on donor and recipient bacterial strains in gut microbiota determine transmission efficiency of blaNDM-1-bearing plasmids. J Antimicrob Chemother.

[28] Yang P, Chen Y, Jiang S, Shen P, Lu X, Xiao Y (2018). Association between antibiotic consumption and the rate of carbapenem-resistant gram-negative bacteria from China based on 153 tertiary hospitals data in 2014. Antimicrob Resist Infect Control.

[29] Kochar S, Sheard T, Sharma R, Hui A, Tolentino E, Allen G (2009). Success of an infectioncontrol program to reduce the spread ofcarbapenem-resistant Klebsiella pneumoniae. Infect Control Hosp Epidemiol.

[30] Abdallah M, Olafisoye O, Cortes C, Urban C, Landman D, Ghitan M (2016). Rise and fall of KPC-producing Klebsiella pneumoniae in New York City. J Antimicrob Chemother.

[31] Harris AD, Pineles L, Belton B, Johnson JK, Shardell M, Loeb M (2013). Universal glove and gown use and acquisition of antibiotic-resistant bacteria in the ICU: a randomized trial. JAMA.

[32] Huskins WC, Huckabee CM, O’Grady NP, Murray P, Kopetskie H, Zimmer L, et al. Intervention to reduce transmission of resistant bacteria in intensive care. N Engl J Med. 2011;364(15):1407–18. 10.1056/NEJMoa1000373 PMC3410743 21488763

[33] Karampatakis T, Tsergouli K, Iosifidis E, Antachopoulos C, Karapanagiotou A, Karyoti A (2018). Impact of active surveillance and infection control measures on carbapenem-resistant Gram-negative bacterial colonization and infections in intensive care. J Hosp Infect.

[34] Schwaber MJ, Lev B, Israeli A, Solter E, Smollan G, Rubinovitch B (2011). Containment of a country-wide outbreak of carbapenem-resistant Klebsiella pneumoniae in Israeli hospitals via a nationally implemented intervention. Clin Infect Dis.

[35] Legeay C, Thépot-Seegers V, Pailhoriès H, Hilliquin D, Zahar JR (2018). Is cohorting the only solution to control carbapenemase-producing Enterobacteriaceae outbreaks? A single-centre experience. J Hosp Infect.

[36] Ben-David D, Maor Y, Keller N, Regev-Yochay G, Tal I, Shachar D (2010). Potential role of active surveillance in the control of a hospital-wide outbreak of carbapenem-resistant Klebsiella pneumoniae infection. Infect Control Hosp Epidemiol.

[37] Zhao ZC, Xu XH, Liu MB, Wu J, Lin J, Li B (2014). Fecal carriage of carbapenem-resistant enterobacteriaceae in a Chinese university hospital. Am J Infect Control.

[38] Liu Q, Liu L, Li Y, Chen X, Yan Q, Liu WE (2019). Fecal carriage and epidemiology of carbapenem-resistant enterobacteriaceaeamong hospitalized patients in a university hospital. Infect Drug Resist.

[39] Qi Y, Wei Z, Ji S, Du X, Shen P, Yu Y (2011). ST11, the dominant clone of KPC-producing Klebsiella pneumoniae in China. J Antimicrob Chemother.

[40] Spencer MD, Winglee K, Passaretti C, Earl AM, Manson AL, Mulder HP (2019). Whole genome sequencing detects inter-facility transmission of carbapenem-resistant klebsiella pneumoniae. J Infect.

[41] Won SY, Munoz-Price LS, Lolans K, Hota B, Weinstein RA, Hayden MK (2011). Emergence and rapid regional spread of klebsiella pneumoniae carbapenemase-producing enterobacteriaceae. Clin Infect Dis.

[42] Gagliotti C, Cappelli V, Carretto E, Marchi M, Pan A, Ragni P (2014). Control of carbapenemase-producing Klebsiella pneumoniae: a region-wide intervention. Euro Surveill.

[43] Fournier S, Monteil C, Lepainteur M, Richard C, Brun-Buisson C, Jarlier V (2014). Long-term control of carbapenemase-producing enterobacteriaceae at the scale of a large French multihospital institution: a nine-year experience, France, 2004 to 2012. Euro Surveill.

[44] Horan TC, Andrus M, Dudeck MA (2008). CDC/NHSN surveillance definition of health care-associated infection and criteria for specific types of infections in the acute care setting. Am J Infect Control.

[45] Queenan AM, Bush K (2007). Carbapenemases: the versatile-lactamases. Clin Microbiol Rev.

[46] Dallenne C, Da Costa A, Decré D, Favier C, Arlet G (2010). Development of a set of multiplex PCR assays for the detection of genes encoding important β-lactamases in enterobacteriaceae. J Antimicrob Chemother.

[47] Brisse S, Passet V, Haugaard AB, Babosan A, Kassis-Chikhani N, Struve C (2013). wzi Gene sequencing, a rapid method for determination of capsular type for Klebsiella strains. J Clin Microbiol.

[48] Walsh TR, Weeks J, Livermore DM, Toleman MA (2011). Dissemination of NDM-1 positive bacteria in the New Delhi environment and its implications for human health: an environmental point prevalence study. Lancet Infect Dis.

